# Assessment of long term outcomes after buccal mucosal graft urethroplasty: the impact of chronic kidney disease

**DOI:** 10.1590/S1677-5538.IBJU.2019.0176

**Published:** 2019-01-29

**Authors:** Ajay Aggarwal, Manoj Kumar, Siddharth Pandey, Samarth Agarwal, Satya Narayan Sankhwar

**Affiliations:** 1 Department of Urology King George’s Medical University Lucknow INDIA Department of Urology, King George’s Medical University, Lucknow, INDIA.

**Keywords:** Oral Mucosal Absorption, Renal Insufficiency, Chronic, Kidney Glomerulus

## Abstract

**Objectives:**

To compare and assess various outcomes and success of buccal mucosal graft urethroplasty (BMGU) in patients with CKD versus patients having normal renal function.

**Material and Methods:**

This was a retrospective, single centre study, during period 2013 to 2017. Patients were grouped into two groups. Group 1 had patients with estimated Glomerular Filtration Rate (eGFR)>60mL/min/1.73m2 while group 2 had patients with eGFR <60mL/min/1.73m^2^. eGFR was calculated according to the MDRD equation. The two groups were compared with regard to various outcomes like length, location of stricture, technique of graft placement, intra-operative blood loss (haemoglobin drop), duration of hospital stay, post-operative complications and recurrence.

**Results:**

A total of 223 patients were included in study with group 1 had 130 patients and group 2 had 93 patients. Mean age of patients with CKD were higher (47.49 years versus 29.13 years). The mean follow-up period was comparable between both groups (23.29 months and 22.54 months respectively). Patients with CKD had more post-operative Clavien Grade 2 or higher complications (p=0.01) and a greater recurrence rates (p<0.001) than in non-CKD patients. On multivariate analysis, age and CKD status was significant predictor of urethroplasty success (p=0.004) (OR= 14.98 (1.952-114.94, 95% CI).

**Conclusions:**

CKD patients are more prone to post-operative complications in terms of wound infection, graft uptake and graft failure and higher recurrence rates following BMGU.

## INTRODUCTION

Urethral stricture is one of the primeval and challenging diseases in urological practice. It may lead to a spectrum of conditions ranging from bothersome lower urinary tract symptoms to Fournier’s gangrene and death. It’s incidence rate is as high as 0.6% in some susceptible populations (1). Corpus spongiosal or urethral epithelial injury may result in urothelial scar formation and stricture disease. The primary aetiologies of urethral stricture disease are idiopathic, trauma, iatrogenic (instrumentation) and infections. Urethroplasty for the management of stricture has been proven highly successful along with its durability and cost-effectiveness (2). End-to-end anastomotic urethroplasty is considered the “gold standard” procedure with 90-95% success rate. But, it is best reserved for bulbar urethral strictures ≤2cm long (3). For more complex strictures, substitution urethroplasty techniques are used with acceptable success rate of approximately 90%. Buccal Mucosal Graft Urethroplasty (BMGU) is a well-established procedure with acceptable results for management of such complex strictures.

Renal impairment affect wound healing due to elevated uraemia toxins which inhibit granulation tissue formation and neovascularisation. This lead to impaired wound healing (4). The success of substitution urethroplasty mainly depend upon proper uptake of graft which may be hampered in patients of Chronic Kidney Disease(CKD). Various studies in literature analysed success of urethroplasty in normal population, but none of them reported outcome in CKD patients. The hypothesis of our study is that patients with normal renal function would have better wound healing and urethroplasty success rate than CKD patients. In our study, our aim was to assess and report the outcome and success of BMGU in moderate and severe chronic kidney disease patients.

## MATERIALS AND METHODS

### We retrospectively analysed our departmental database

Inclusion criteria: Patients who underwent BMGU between January 2013 to December 2017 Exclusion criteria: Patients who had history of previous perineal surgical procedure, radiotherapy and having multiple strictures.

In the above mentioned period, 252 patients underwent BMGU, out of which 223 met inclusion criteria. Patient were grouped into CKD patients (Grade 3 or higher, KDIGO criteria (5)) (eGFR <60mL/min/1.73m^2^) and non-CKD (eGFR >60mL/min/1.73m^2^) patients using MDRD equation (6). Approval was taken from the institutional ethical committee before study (1443/Ethics/R.cell-18).

Patient’s age, eGFR, duration of presentation, other comorbidities like diabetes and hypertension, addiction like smoking, previous procedures like Optical Internal Urethrotomy (OIU) or Sequential Dilation (SqD), location, length, site and aetiology of stricture, post-operative haemoglobin drop, hospital stay after surgery were recorded. Post-operative complications (Clavien-Dindo Classification (CDC)) were also recorded.

## SURGICAL PROCEDURE

All the surgeries were performed by two consultants of the urology department (MK and SNS), well versed with the technique used. All surgeries were carried out under regional anaesthesia. There were two teams involved in most surgeries: one for harvestation of buccal mucosa and the other team for perineal dissection. Buccal mucosa graft was harvested under local anaesthesia (7). Dorsal/dorso-lateral onlay BMGU was carried out using technique described by Singh et al. (8) whereas ventral onlay BMGU was done using technique described by Wessels (9).

Prophylactic intravenous antibiotic (third generation cephalosporin or ampicillin and gentamicin) was given at time of induction of anaesthesia. The oral pack was removed in the evening of the day of surgery and cold liquids allowed orally. First dressing of perineal site was done 48 hrs after surgery and then daily thereafter. Patients were discharged with Foley in situ and oral antibiotics (fluoroquinolones) were prescribed till catheter was in situ. Foley catheter was removed after 4-6 weeks.

## FOLLOW-UP

In follow-up, clinical history and uroflowmetry with post void residual urine were performed at 3-month intervals for initial year and then yearly thereafter. Urethroplasty success was defined as Qmax greater than 15mL/sec. Urethroplasty failure/recurrence was declared when post-operative intervention was needed such as OIU/SD or redo-urethroplasty due to obstructive symptoms reported by patient followed by abnormal urethrogram/urethroscopy.

## DEFINITION OF CKD

Estimated glomerular filtration rate (eGFR) was calculated using MDRD equation (eGFR/mL/min/1.73m^2^=186*[SCr]-1.154*[age]-0.203*[0.742 for female]). As per National Kidney Foundation KDOQI TM, CKD was defined as eGFR <60mL/min/1.73m^2^ (10).

## STATISTICAL ANALYSIS

Data were compiled and entered on MS Office Excel 2016 spreadsheet. Analysis was done using SPSS (version 23.0; IBM, USA). The paired t-test was used for continuous variables, to detect the level of significance between baseline and follow-up data. The unpaired t test was used to detect the difference between the two intervention arms. The categorical data were analysed by Fisher’s exact test. P <0.05 was considered significant.

## RESULTS

Patients were categorised into two groups. Group-1 contained patients with eGFR more than and equal to 60mL/min/1.73m^2^ and Group-2 had patients with eGFR less than 60mL/min/1.73m^2^. The mean age of patients in group-2 was significantly higher (47.49±11.71 years) than in group-1 (29.13±12.39 years) (p <0.001). Patients with CKD presented early (17.68±12.13 months) as compared with non-CKD patient (23.97±16.56 months) (p=0.01). The mean follow-up period in groups-1 and 2 was 23.29 months and 22.54 months respectively. Most of strictures in both groups were idiopathic in nature (67.7% and 57% respectively). Other aetiologies of stricture and previous procedures like optical internal urethrotomy (OIU)/sequential dilation (SqD) in both groups are shown in Table-1.

**Table 1:** Patient characteristics and demographic profile.

	eGFR>60(n=130)	eGFR<60 (n=93)	P value
**Pre-operative parameters**			
Age(years)(mean ± SD)	29.13 ± 12.39	47.49 ± 11.71	**<0.001**
BMI(kg/m^2^)	23.21 ± 3.23	22.78 ± 4.12	0.76
Duration of presentation(months) (mean ± SD)	23.97 ± 16.56	17.68 ± 12.13	**0.01**
Follow up period(months) (mean ± SD)	23.29 ± 8.16	22.54 ± 6.14	0.45
**Co-morbidities**			
Diabetes(%)	11(8.46)	10(9.3)	-
Hypertension(%)	10(13.0)	40(43.0)	-
**Etiology of stricture**			
Traumatic(%)	7(5.3)	0(0)	**0.02**
Iatrogenic(%)	27(20.8)	40(43)	**<0.001**
Idiopathic(%)	88(67.7)	53(57)	0.10
Inflammatory(%)	8(6.2)	0(0)	**0.01**
**Previous procedure**			
(OIU)/ (SqD) (%)	46(35.4)	25(26.9)	0.17
**Intraoperative parameters**			
Length of stricture(cm) (mean ± SD)	3.85 ± 1.52	4.19 ± 1.47	0.10
<2cm, n(%)	26(20)	3(3.2)	-
2-4cm, n(%)	62(47.7)	56(60.2)	-
>4 cm, n(%)	42(32.3)	34(36.6)	-
**Location of stricture**			
Pan anterior(%)	24(18.5)	8(8.6)	**0.03**
Bulbar/ penobulbar(%)	106(81.5)	85(91.4)	
Graft Placement			
Dorsal/Dorso-lateral onlay(%)	111(85.4)	60(64.5)	-
Ventral onlay(%)	19(14.6)	33(35.5)	-
**Post-operative parameters**			
Haemoglobin drop (gm%)(mean ± SD)	1.26 ± 0.73	1.44 ± 0.66	0.054
Hospital stay(days) (mean ± SD)	9.4 ± 4.08	9.09 ± 1.71	0.438
**Clavien Dindo**			
0(%)	109(83.9)	44(47.3)	**0.07**
1(%)	9(6.9)	2(2.1)	0.22
≥ 2(%)	12(9.2)	47(50.5)	**0.01**
Recurrence(%)	6 (4.6)	37(39.78)	**< 0.001**
**Recurrence in stricture ,n (%)**			
<2cm	0(0)	0(0)	**-**
2-4cm	6(100)	20(54)	**<0.001**
>4cm	0(0)	17(46)	<0.001

**eGFR** = estimated Glomerular Filtration rate; **BMI =** Body Mass Index; **SD =** Standard deviation; **OIU =** Optical Internal Urethrotomy; **SqD =** Sequential Dilation.

Intra-operatively, majority of strictures in both groups were bulbar/penobulbar in location versus pan-anterior urethral strictures (Table-1). The mean length of stricture in groups 1 and 2 was 3.85±1.52 and 4.19±1.47cm respectively (p=0.10). Around 85% patients in group 1 and 65% patients in group 2 had dorsal/dorso-lateral placement of buccal mucosal graft. In rest of patients, ventral onlay placement was done. Post-operative haemoglobin drop was more in CKD patients (1.44±0.66gm/dL) as compared in non-CKD patients (1.26±0.73gm/dL), but there was no statistical difference (p=0.054). The mean length of hospital stay was also comparable between both groups (Table-1).

83.9% and 47.3% patients in group 1 and group 2 respectively had no complications (Clavien-Dindo Classification, CDC-0) post-operatively, (p=0.07, OR=0.59 (0.33-1.05),95% confidence interval). CKD patients had significantly more CDC ≥2 complications than in non-CKD patients and were statistically significant (p=0.01). Also, urethroplasty failure/recurrence rate was significantly higher in CKD patients, (p <0.001) (Table-1). On multivariate regression analysis for risk factors associated with stricture recurrence, age and CKD were found to be significantly associated with stricture recurrence (Table-2).

**Table 2:** The multivariate logistic regression analysis for risk factors associated with stricture recurrence.

Risk factor	P value	OR (95% CI)
Age	**0.029**	1.067(1.003-1.135)
Pan Anterior	0.125	0.238(0.019-2.914)
Bulbar/Penobulbar	0.432	0.642(0.212- 0.988)
CDC-0	0.298	0.243(0.023-2.615)
CDC-≥ 2	0.083	6.431(0.614-67.36)
CKD	**0.004**	14.98(1.952-114.94)

**CDC** = Clavien- Dindo Complication; **CKD** = Chronic Kidney Disease

## DISCUSSION

Urethral stricture management is a demanding surgery in urological field. Before the emergence of newer techniques using flaps and grafts, the results of management of long-term strictures were poor. In 1972, Orandi successfully used penile skin flap in one stage urethroplasty for anterior urethral strictures (11). Humby et al. in 1941 demonstrated use of buccal mucosal graft for urethral construction (12). Nowadays, buccal mucosa is considered as ideal urethral substitute for reconstruction due to its ease of harvesting, wet environment and early ingrowth and graft survival.

CKD is defined as a persistent reduction in GFR to below 60mL/min/1.73m^ ^ for three months or the presence of haematuria, proteinuria/microalbuminuria and radiologic/histologic changes in the kidneys. Renal impairment has been known to affect wound healing. Heller et al. demonstrated association of chronic kidney disease with impaired wound healing and as an independent risk factor for incisional hernia development (13). Research data on mice also showed deteriorating effect of CKD on wound healing. This is mediated by the disruption of keratinization mechanics, delayed granulation, and large epithelial gap. The veiled chronic inflammatory state and low rate of neovascularization and cellular proliferation also contributed to poor wound healing (14, 15).

In our study, mean age of patients who had CKD was significantly higher than non-CKD patients (47.49 year’s vs. 29.13 years, p <0.001). This was mostly caused by diabetes and hypertension, which are most common causes of chronic kidney disease worldwide and more prevalent in elderly age group. Different studies in literature evaluated various predictors of urethroplasty success. One such study done by Singh et al. concluded that age of patient do not predict outcome after urethroplasty (16). However, in our study, on multivariate logistic regression analysis for risk factors associated with stricture recurrence, age was a significant factor (p=0.029), OR=1.067 (1.003-1.135, 95% CI) (Table-2). Various studies in literature mentioned obesity as a predicting factor for stricture recurrence (17, 18). In our study both groups had comparable body mass index.

Patients with normal GFR had longer duration of presentation of stricture (23.97+16.56 months) that was statistically significant as compared with CKD patients (17.68+12.13 months, p=0.01). The most possible explanation for this finding is that CKD patients have more frequent hospital visits and check-ups, during which stricture might be detected early.

In our study, patients with hypertension were more in number in group 2, owing to the likely impact of HTN on eventual CKD. However, diabetic patients were almost equal in both groups. Our study was under-powered to assess the impact of HTN and DM as independent variable on the success and outcomes of BMGU in both groups.

Almost 90% of strictures in non-CKD patients and 100% in CKD patients, were idiopathic or iatrogenic in nature. Stein et al. (19) looked retrospectively at 2.589 patients who underwent urethroplasty procedures from 2000 to 2011 in the USA, Italy, and India. They also concluded similar results regarding aetiology of stricture as found in our study. The reason for higher percentage of iatrogenic strictures in CKD patients (43%) as compared to non-CKD patients (20.8%) is not known. Almost 36% and 27% patients in group 1 and group 2 respectively had previous history of surgical procedures (OIU/SqD). Singh et al. (16) studied 58 patients with post-traumatic posterior urethral stricture who underwent anastomotic urethroplasty. They concluded that previous failed procedures significantly decrease the success of subsequent anastomotic urethroplasty. In another study, Barbagli et al. (20) retrospectively reviewed a series of 93 patients comparing patients with primary repair versus patients with prior history of urethrotomy and underwent secondary treatment. They concluded that failed urethrotomy does not affect the long-term result of surgical repair. In our study, both groups were comparable in terms of previous procedures.

Long length of strictures(>4cm) are usually associated with increased risk of recurrence (21). In our study, mean length of stricture in both groups were comparable (3.85+1.52 vs. 4.19+1.47cm respectively, p=0.10). Around one third of patients had stricture >4cm in both groups (Table-1). Patients with CKD had more number of patients with bulbar/peno-bulbar strictures (p=0.03) in comparison with pan-urethral strictures. The reason for this finding is unknown. Meeks et al. (22) described surgical outcomes in patients with kidney and kidney-pancreas transplants after urethroplasty for stricture or fistula disease. Around 80% patients in that study had mid-bulbar/peno-bulbar strictures and only 20% had penile urethral strictures.

Various techniques of BMGU have been mentioned in literature, including dorsal onlay, ventral onlay, lateral onlay and combined techniques, all having success rates above 90%. Among all, dorsal/dorso-lateral onlay and ventral onlay techniques are the most commonly used (23, 24). In our study, most of the cases in both groups (85.4% and 64.5% respectively) used dorsal/dorsolateral onlay technique followed by ventral onlay technique depending on the site of stricture and surgeon discretion. Pathak et al. (25), conducted retrospective analysis on 112 patients who underwent BMGU for non-traumatic long segment bulbar urethral strictures. They compared long- term outcomes of BMGU by placing the graft ventrally, dorso-laterally and dorsally. The author found that the overall success rate for BMG augmentation urethroplasty is equal for all techniques. Other studies reported similar success rates of 85-97% and 83-94% for dorsal and ventral BMGU, respectively (26, 27). In our study, patients with CKD had significantly more ventral onlay placement of buccal mucosa and non-CKD patients had more of dorsal/dorsolateral graft placement (Table-1). However, the decision of graft position was solely based on surgeon’s choice.

Patients with CKD are more prone for blood loss during any surgery. Platelet dysfunction is the main factor responsible. Various factors like anaemic state of patient, dialysis per se, delayed clearance of medications and anticoagulation used during dialysis also play some role in causing impaired haemostasis in these patients. Platelet dysfunction occurs due to impaired platelet aggregation and impaired platelet-vessel wall interaction. The normal platelet response to vessel wall injury include platelet activation, recruitment, adhesion, and aggregation which are impaired in chronic renal failure. A systematic review and meta-analysis done by Acidello et al. (28), screened 9376 citations from multiple databases, concluded that chronic kidney disease is associated with significant perioperative bleeding but rarely required reoperation. In our study, mean haemoglobin drop in CKD patients was higher, however, statistically comparable with patients having normal renal function (1.26±0.73 and 1.44±0.66g/dL respectively, p=0.054).

Both groups of patients had statistically comparable length of hospital stay (9.4±4.08 and 9.09±1.71days, p=0.43). Patients in our study usually come from far areas to our tertiary care centre and they prefer to stay in hospital till their suture removal, explaining the long length of stay in hospital.

Around 84% patients in non-CKD group had no post-operative complications (Clavien-Dindo=0) as compared to only 47% patients in CKD group. We also observed significantly higher CDC ≥2 complications in patients with CKD (50.5% vs. 9.2%, p=0.01) (Table-1). These complications included surgical site infection, urinary fistula, graft contracture and graft failure. In patients with stricture length less than 2cm, none of them had recurrence (Table-1). Patients with stricture length between 2 to 4cm and more than 4cm had significantly higher recurrence rate in group-2. It implied that stricture length did not affect BMGU success. Overall recurrence rate was also significantly higher (4.6% vs. 39.78%, p=<0.001) in CKD patients versus patients with normal renal function on bivariate analysis. On multivariate analysis also, CKD appeared as significant risk factor for stricture recurrence (p=0.004) (OR=14.98 (1.952-114.94, 95% CI) (Table-2). Mori et al. (29) reported an overall complication rate of 19.2% and a revision rate of 10.3% in 78 patients who underwent multistage reconstruction for complex anterior urethral strictures. The revision rate in contemporary series ranges from 6.7% to 59% in patients with normal renal function, and our results are in line with literature (30). The possible reason for more complications and urethroplasty failure rate in CKD patients is due to delayed vascularisation and graft uptake leading to delayed wound healing and ultimately graft failure (15).

The strength of our study lies on its simplicity and the fact that in best of our knowledge, till date, no study in literature compared different outcomes following BMGU between CKD patients and patients with normal renal function.

Limitations of our study include: (i) retrospective analysis (ii) follow-up limited to 30 months. Longer follow-up is required to determine if stricture recurrence rates between the two groups changes over time. (iii) Data on patients having haemodialysis is lacking.

## CONCLUSIONS

Buccal mucosal graft urethroplasty is an established procedure for stricture urethra with overall success rate of around 94%. However, in patients with CKD, overall success rate drops down to 70% with more post-operative complications. Comorbidities like diabetes, length, location, duration of stricture and technique of graft placement do not affect outcomes in CKD patients.
